# Virginal Breast Hypertrophy: A Case Report

**DOI:** 10.7759/cureus.40067

**Published:** 2023-06-06

**Authors:** Sarah A Soliman, Mohammad A Algatheradi, Thuraya A Aljahwashi, Taef H Alhussan, Riyadh S Alqahtani, Nahid I Ali

**Affiliations:** 1 Diagnostic Radiology, Military Hospital in Southern Region, Abha, SAU; 2 Diagnostic Radiology - Pediatric, Abha Maternity and Children Hospital, Abha, SAU; 3 Women Imaging Radiology, Aseer Central Hospital, Abha, SAU; 4 Radiology, Armed Forces Hospitals, Abha, SAU; 5 Plastic Surgery, Abha Maternity and Children Hospital and Aseer Central Hospital, Abha, SAU; 6 General Radiology, Abha Maternity and Children Hospital, Abha, SAU

**Keywords:** case report, virginal mammary hypertrophy, juvenile gigantomastia, juvenile macromastia, virginal hypertrophy

## Abstract

Virginal breast hypertrophy, also known as juvenile macromastia or juvenile gigantomastia, is an uncommon condition characterized by the rapid and excessive growth of breasts in prepubertal or peripubertal girls in the absence of any hormonal or physiological causes. While virginal breast hypertrophy is a rare benign disorder that occurs independent of hormonal stimulation, it can cause a diagnostic challenge to physicians and requires a multidisciplinary team to get it right. It also results in detrimental effects, both physical and psychological, for young girls. We present a case of virginal breast hypertrophy in an 11-year-old Saudi girl, which was successfully managed. This report will contribute to knowledge sharing with healthcare professionals in Saudi Arabia about this rare case. It can also pave roads for further research to understand the underlying mechanisms and to standardize treatment modalities.

## Introduction

Breast hypertrophy, defined as excessive enlargement of breast tissue, is a condition that can affect women of various age groups [1]. This condition, also known as juvenile macromastia or juvenile gigantomastia can manifest as either a congenital anomaly or develop during puberty. However, in rare cases, breast hypertrophy can occur in prepubertal or peripubertal young women with no previous history of breast development. This atypical presentation is referred to as "virginal breast hypertrophy” [2-3], and is defined as breast weight exceeding 3% of the total body weight or breast tissue weight exceeding 1.5-1.8 kg [4]. It can be unilateral, bilateral, symmetrical, or asymmetric.

While most cases of breast hypertrophy occur during puberty or following pregnancy, virgin breast hypertrophy presents an intriguing clinical scenario. It involves the sudden and spontaneous enlargement of breasts in young women who have not previously experienced any breast development or hormonal changes [4]. The exact etiology of virginal breast hypertrophy remains largely unknown. Hormonal imbalances, genetic predispositions, and idiopathic factors have been proposed as potential contributors [3].

The clinical presentation of virginal breast hypertrophy typically involves a rapid increase in breast size, leading to physical discomfort and functional limitations. The breasts' rapid growth results in skin stretches, the loss of areola definition, and ulcerations [4]. Patients often experience symptoms such as breast pain, neck and back pain, shoulder grooving from bra straps, difficulty in finding properly fitting clothing, and impaired physical activities. Psychosocially, it may result in body image dissatisfaction, low self-esteem, and emotional distress [2-3].

Diagnosing virginal breast hypertrophy requires excluding other possible causes of breast enlargement, such as hormonal abnormalities, tumors, or cystic lesions [5]. Additionally, psychological assessments may be necessary to assess the emotional impact of the condition on the patient [6].

Conservative treatment options are inadequate for addressing the functional and psychological implications of the condition, and breast reduction surgery is considered the most effective and long-lasting treatment for virginal breast hypertrophy [4, 6].

We will present a successfully treated virginal breast hypertrophy case to share insights gained from managing this rare case with other healthcare professionals who may receive similar cases.

## Case presentation

**Figure 1:**
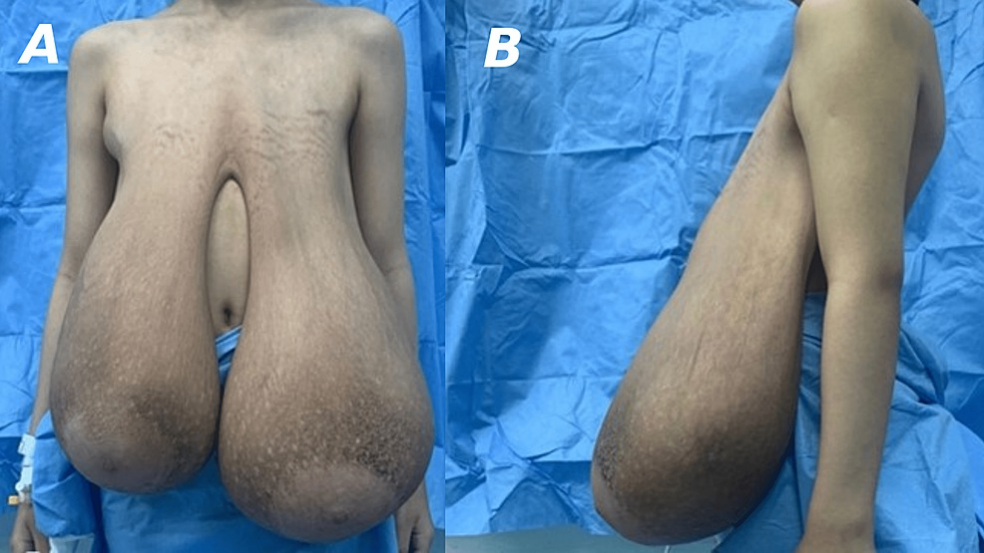
Preoperative anterior (A) and lateral (B) views showing breast enlargement.

**Figure 2:**
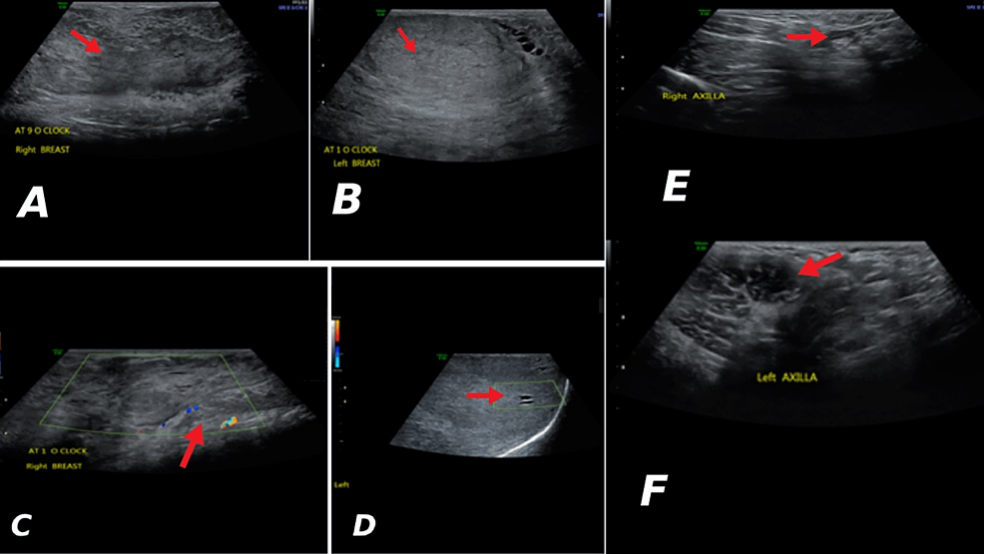
Heterogenous parenchymal echotexture predominantly hyperechoic without definite masses in both right (A) and left (B) breasts and the color doppler images of right (C) and left (D) breasts. No suspicious enlarged axillary lymph nodes in both breasts (E and F).

**Figure 3:**
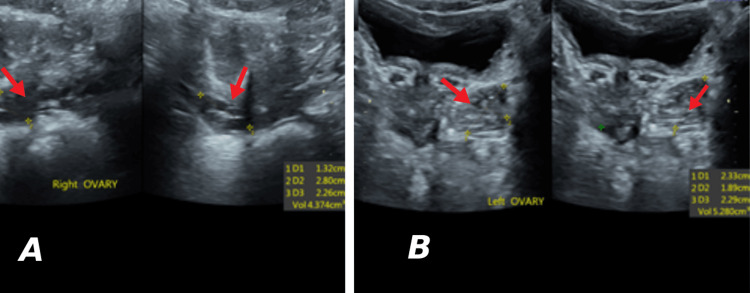
Ultrasound images showing ovaries with small follicles. A: Right ovary measuring 4.3 cm^3^; B: Left ovary measuring 5.2 cm^3^

An 11-year-old Saudi girl presented in February 2023 with complaints of painless rapid breast enlargement within four months. The patient had no significant medical history, and her parents reported no family history of breast cancer or similar conditions. She was not taking any medications, and her first menstrual cycle came after breast enlargement in April 2023 and lasted for six days. Physical examination revealed markedly enlarged breasts with skin stretching and striae, significantly disproportionate to her age (Figure 1). However, palpation revealed no tenderness, no palpable masses, or signs of inflammation or nipple discharges. The remainder of the physical examination was unremarkable. Under aseptic measurement, a true-cut biopsy,

Laboratory tests, including hormone levels, such as estradiol (E2), progesterone (P4), luteinizing hormone (LH), follicle-stimulating hormone (FSH), prolactin (PRL), and thyroid stimulating hormone (TSH) were within normal limits. A breast ultrasound was performed, confirming the presence of hypertrophic mammary glands with increased breast tissue, with a dilated duct system interspersing tiny cystic areas, but no definite masses (Figure 2). Transabdominal showed a mild increase in left and right ovarian volumes (4.3 cm^3^ and 5.2 cm^3^, respectively) for her age (Figure 3).

**Figure 4:**
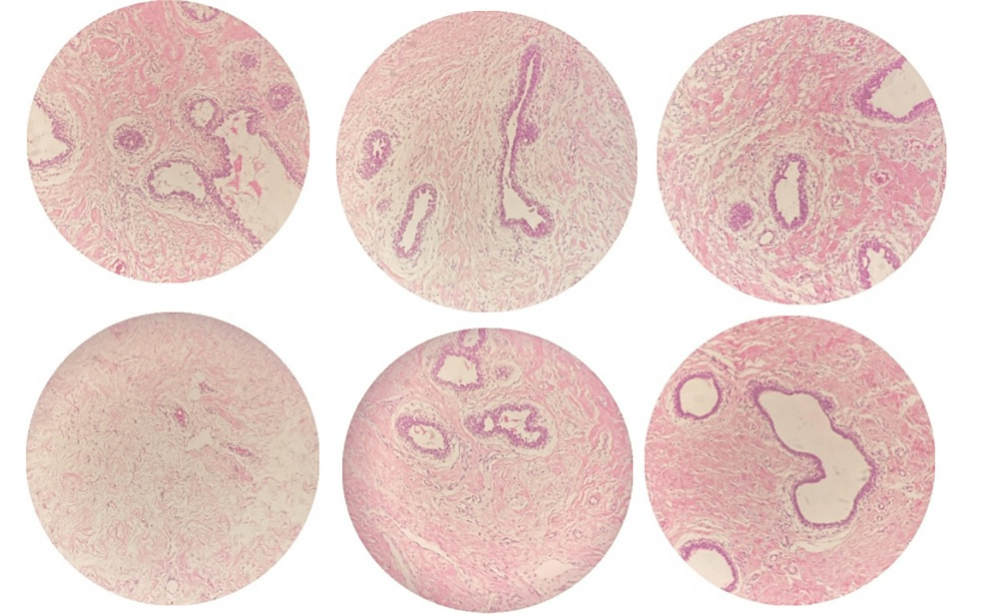
Features of biphasic proliferating lesion composed of benign hyperplastic multilayered ducts in densely collagenous to fibrous stroma containing dilated vascular channels, and no atypia or evidence of malignancy seen.

**Figure 5:**
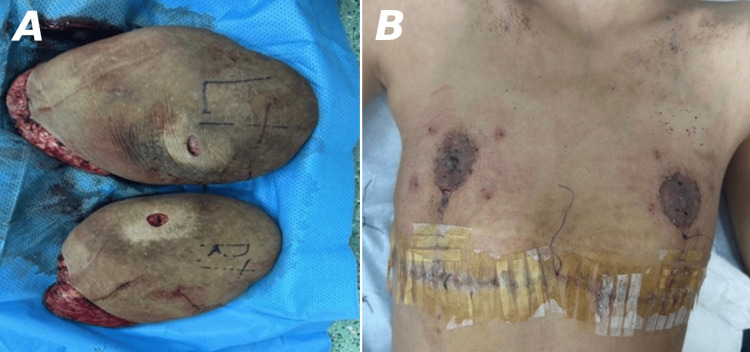
Excised breast tissues (A) and an image showing a surgical scar three days post-surgery (B).

Under aseptic conditions, a true-cut biopsy was done and histopathological results showed a biphasic proliferating lesion composed of benign hyperplastic multilayered ducts in densely collagenous to fibrous stroma containing dilated vascular channels without evidence of atypia or malignant cells (Figure 4), consisting of virginal hypertrophy. Then, she underwent a reduction mammoplasty with a free nipple areola graft, and excised breast tissues were sent for post-operatively, the patient was stable with clean wounds (Figure 5), and she was discharged after 6 days with a follow-up appointment in 1 week.

## Discussion

Hormonal imbalance, genetics, and local factors are thought to contribute to the development of virginal breast hypertrophy [3]. Early diagnosis and appropriate management are crucial to address physical discomfort, to prevent functional impairment, and to minimize psychological distress associated with the condition [7]. The etiology of virginal breast hypertrophy remains unclear, and managing this condition poses several challenges due to its rarity and varied clinical presentations [8]. The first case of virginal breast hypertrophy in medical literature was reported in 1669 in UK [9-10]. Virginal breast hypertrophy is so rare that over 100 years from 1910 to 2009, only 65 cases had been reported in the medical literature [11], presents among girls aged between 10 and 17 [9, 12].

Virginal breast hypertrophy typically manifests during early adolescence, when hormonal changes occur [6]. Hormones such as estrogen, progesterone, and prolactin influence breast development, and an imbalance in their levels is thought to lead to excessive breast growth [13]. Genetic factors may also contribute to the development of virginal breast hypertrophy, as reported cases of this condition occur within families [3]. Research has suggested that mutation and deletion of the PTEN gene are associated with early lobuloalveolar development, excessive ductal branching, delayed involution, reduced apoptosis, and mammary epithelial hyperproliferation [14-15]. The primary symptom is the rapid enlargement of one or both breasts, which often occurs over weeks or months. Excessive breast size can cause physical discomfort, pain, and difficulty with movement and posture. Additionally, affected individuals may experience emotional distress, body image issues, and low self-esteem [6, 13]. Diagnosing involves a thorough medical evaluation and imaging techniques like mammography, ultrasound, or MRI to rule out other causes, such as tumors or hormonal disorders [16], including giant fibroadenomas, phyllodes tumors, and malignant tumors, which are common in adolescents with breast lesions [3].

In cases where the breast enlargement is mild and not causing significant discomfort, conservative management, such as well-fitted bras and engaging in exercises that improve posture and strengthen the back muscles, may be sufficient [13]. However, for individuals with severe symptoms, surgical intervention is often necessary [4, 6]. Breast reduction surgery, or reduction mammoplasty, is the most common surgical procedure to remove excess breast tissue and reshape the breasts to a more proportionate size, alleviating physical discomfort, improving mobility, and enhancing the individual's self-esteem and body image [4, 6]. The surgical options include reduction mammoplasty (pedicle-based or with a free nipple graft) and mastectomy with implant reconstruction. However, reduction mammoplasty was found to be seven times more likely to lead to recurrence (p=0.01), making mastectomy the most effective intervention [11]. It is recommended to minimize the use of mastectomy with implant reconstruction in adolescents due to the procedure's length and lifelong implant reconstruction risk, which has potential psychological effects related to self-image and psychosocial development. Therefore, reduction mammoplasty should be used as the first-line intervention, followed by mastectomy in the case of recurrence [3]. Thus, our patients underwent a reduction mammoplasty with a free nipple areola graft. It was indicated that a free nipple graft significantly leads to less risk of recurrence than a pedicled technique (p = 0.005) [17]. Postoperative care following breast reduction surgery involves monitoring for complications, such as infection, bleeding, or scarring, and ensuring appropriate wound healing.

## Conclusions

Virginal breast hypertrophy is a rare condition characterized by excessive breast growth in adolescent girls. Although the exact causes are not fully understood, hormonal imbalances are believed to play a significant role. The condition can have profound physical, emotional, and psychosocial consequences for affected individuals. Diagnosis involves a thorough medical evaluation, including imaging studies, to rule out other causes of breast enlargement. Treatment options are conservative management and surgical intervention, with surgery being the more effective. The patients’ management modalities should address the physical and psychological aspects of this condition.
